# Long Noncoding RNA LOC100129940-N Is Upregulated in Papillary Thyroid Cancer and Promotes the Invasion and Progression

**DOI:** 10.1155/2019/7043509

**Published:** 2019-04-07

**Authors:** Manting Choy, Yan Guo, Hai Li, Guohong Wei, Runyi Ye, Weiwei Liang, Haipeng Xiao, Yanbing Li, Hongyu Guan

**Affiliations:** ^1^Department of Endocrinology and Diabetes Center, The First Affiliated Hospital of Sun Yat-sen University, China; ^2^Department of Thyroid and Breast Surgery, The First Affiliated Hospital of Sun Yat-sen University, China

## Abstract

Thyroid cancer is the most common endocrine malignancy, and its incidence has increased rapidly in recent decades worldwide. Papillary thyroid cancer (PTC) is the most common type of all thyroid cancers. The molecular mechanisms underlying the disease still need to be further investigated. Long noncoding RNAs (lncRNAs), a class of noncoding RNAs (ncRNAs) longer than 200 nucleotides, are aberrantly expressed in malignant diseases, including PTC. Here, we identified a novel isoform of LOC100129940 and designated it as LOC100129940-N. We demonstrated that the expression level of LOC100129940-N was elevated in PTC, indicating that LOC100129940-N may be involved in PTC development and progression. Moreover, our results showed that overexpression of LOC100129940-N promoted, whereas silencing of LOC100129940-N suppressed, PTC cell proliferation, invasion, and migration. Mechanistically, LOC100129940-N played an important role in activating Wnt/*β*-catenin signaling and upregulating downstream target genes. Taken together, we demonstrate that LOC100129940-N promotes the activation of Wnt/*β*-catenin signaling, which in turn regulates the downstream target genes, thereby enhancing invasion and progression of PTC.

## 1. Introduction

Thyroid cancer is the most common endocrine malignancy, and its incidence has increased rapidly in recent decades worldwide [1, 2]. Thyroid cancer consists of several histological variants, and papillary thyroid cancer (PTC) is the most common type, accounting for about 80% of all thyroid cancers [3, 4]. In most cases, PTC is highly curable. However, some patients have an aggressive disease course [5]. The molecular mechanisms underlying the aggressive disease course still need to be further investigated.

Long noncoding RNAs (lncRNAs) are a class of noncoding RNAs longer than 200 nucleotides [6]. Accumulating evidence has demonstrated that lncRNAs are poorly conserved and play critical roles in regulating various cellular biological processes, such as Th-cell differentiation [7], autophagy [8], and senescence [9], by affecting a wide range of aspects of protein and DNA, as well as RNA expression and interactions. The aberrant expression of lncRNAs has been found in multiple cancers, including PTC [10, 11]. lncRNAs can regulate tumor suppressors or oncogenes via different mechanisms including RNA decoy, alternative splicing, and epigenetic, transcriptional, and posttranscriptional modification [12, 13]. For example, the elevation of lncRNA HOTAIR in breast cancer promotes ligand-independent estrogen receptor (ER) activities, thereby contributing to tamoxifen resistance [14]. Moreover, a study by Kelly et al. showed that lncRNA HIT000218960 promoted PTC cell proliferation via regulating the expression of HMGA2 [15]. It has been demonstrated that the expression of LOC100129940 (lnc-LLPH-2:1, lnc-RP11-366L20.2.1-2:23, ENST00000504038.2, and NR 120478.1) was upregulated in PTC, suggesting the potential role of LOC100129940 in PTC [16]. Here, we discovered a novel isoform of LOC100129940 and designated it as LOC100129940-N. Its clinical significance, biological function, and underlying mechanisms were identified.

Wnt/*β*-catenin signaling is involved in various processes that are pivotal for cancer development and progression, including cancer initiation, growth, invasion, and metastasis [17–19]. Without Wnt stimulation, cytoplasmic *β*-catenin is degraded by the destruction complex, whose components include Axin, adenomatous polyposis coli (APC), glycogen synthase kinase-3 (GSK3), and casein kinase 1 (CK1). In this complex, *β*-catenin is phosphorylated and subsequently destructed by the proteosomal machinery [20, 21]. Binding of Wnt to frizzled (FZD) receptors and low-density lipoprotein (LDL) receptor-related protein 5/6 (LRP5/6) coreceptors results in a series of events that disrupt the destruction complex [22, 23]. This stabilizes *β*-catenin and increases its nuclear translocation, resulting in the regulation of downstream target genes [24]. Accumulating evidence has demonstrated that Wnt/*β*-catenin signaling also plays an important role in PTC [25–27]. Further understanding of the regulatory network of Wnt/*β*-catenin signaling in PTC will put new insights into the etiology of the disease.

## 2. Materials and Methods

### 2.1. Cell Lines

The human PTC cell line KTC1 was purchased from the Cell Bank of Type Culture Collection of Chinese Academy of Sciences (Shanghai, China). TPC1 (human PTC cell line) was kindly provided by Prof. Haixia Guan (China Medical University, China). KTC1 and TPC1 cell lines were characterized and authenticated using short tandem repeat (STR) DNA profiling. The cells have been validated to be mycoplasma-free. KTC1 and TPC1 cells were incubated in Dulbecco's modified Eagle's medium (Gibco Laboratories, Grand Island, NY, USA) supplemented with 10% fetal bovine serum (FBS, Gibco Laboratories) at 37°C and 5% CO_2_.

### 2.2. Patients and Tissue Specimens

A total of 59 cases of fresh PTC and 11 pairs of fresh PTC and their matching noncancerous tissues were obtained from the First Affiliated Hospital of Sun Yat-sen University between 2015 and 2017. Three normal thyroid tissues were obtained from donors who died in traffic accidents, and none of them has detectable pathological conditions. All specimens were histopathologically and clinically diagnosed by two pathologists. Written informed consent was obtained from patients for use of the samples. Ethical approval was obtained from the Institutional Research Ethics Committee.

### 2.3. 5′ and 3′ Rapid Amplification of cDNA Ends (RACE) Analysis

The transcriptional initiation and termination sites of LOC100129940-N were determined by 5′ and 3′ RACE-polymerase chain reaction (PCR) using the SMARTer RACE 5′/3′ Kit (TaKaRa, Tokyo, Japan), according to the supplier's protocol. Primers used for the RACE-PCR analysis are as follows: 5′ RACE, 5′-TCTGGGTTGCTGGGTGCTTTCTTTG-3′, and 3′ RACE, 5′- CACCTCACTGACTGCTCACCTGT-3′.

### 2.4. RNA Extraction, Reverse Transcription (RT), and Quantitative RT-PCR (qRT-PCR)

Total RNA of cultured cells or fresh tissues was extracted using TRIzol (Invitrogen). The first-strand cDNA was obtained using Moloney murine leukemia virus reverse transcriptase (MMLV, Promega, Madison, WI) with random primers. qRT-PCR was performed using SYBR©Green PCR Master Mix (Roche Diagnostics, Mannheim, Germany) on a CFX96 real-time PCR detection system (Bio-Rad, Richmond, CA). GAPDH was used as an internal control to normalize the indicated genes. Each sample was analyzed three times in triplicate. Data are calculated by the 2^−ΔΔCt^ method. The sequences of all primers for the indicated genes are listed in Supplementary Table 1.

### 2.5. Cell Nucleus/Cytoplasm Fraction Isolation

A commercial cell fractionation kit (Nuclear and Cytoplasmic Extraction Kit, Thermo, Waltham, MA) was used to isolate nucleus/cytoplasm RNA extracts in accordance with the manufacturer's instruction.

### 2.6. Plasmid Construction and Retroviral Infection

The full length of LOC100129940-N was obtained by PCR and cloned into the retroviral expression vector pQCXIP (Clontech, Mountain View, CA). We used empty pQCXIP as a vector control. The indicated plasmids were transfected into packing cells (PT67, Clontech), an NIH3T3-derived cell line expressing the 10Al viral envelope, using Lipofectamine 3000 (Invitrogen). The supernatant from the transfected PT67 cells was then harvested and passed through a 0.45 *μ*m filter. Subsequently, the supernatant was incubated with the indicated cells in the presence of 8 *μ*g/ml polybrene. For the selection of stable cell lines, the cells were cultured with 0.5 *μ*g/ml of puromycin for 14 days [28].

### 2.7. Transfection of lncRNA Smart Silencer

Specific lncRNA Smart Silencer for LOC100129940-N was designed and synthesized by RiboBio (Guangzhou, China). LOC100129940-N Smart Silencer is a mixture of three siRNAs and three antisense oligonucleotides (ASOs). The target sequences of siRNAs are as follows: 5′-CCATTGGAATCTCCTCTCA-3′, 5′-ACAGTACCATTCTAGCAAA-3′, and 5′-CCACAGGGCAAATAAAGCT-3′. The target sequences of ASOs are as follows: 5′-CAGCTCACATCAAGTCACCG-3′, 5′-CCATTATGTCTCCCAGTTCT-3′, and 5′-TCCACGTCAACATTCCACCC-3′. The negative control (NC) Smart Silencer does not contain domains homologous to humans, mice, and rats. Transfection was performed using Lipofectamine 3000 (Invitrogen), in accordance with the manufacturer's instructions.

### 2.8. Transwell Assay

Transwell invasion and migration assays were performed as previously described [29]. Briefly, 2 × 10^4^ indicated cells suspended in 200 *μ*l of serum-free medium were added to the apical chamber of Transwell inserts (Millipore, Bedford, MA, USA) precoated with or without Matrigel (BD, Bedford, MA, USA). Then, the inserts were placed in 24-well plates containing medium with 10% FBS. After 24 h incubation, the noninvading and nonmigrating cells on the upper side of the inserts were removed by wiping with cotton swabs. Subsequently, the migrated and invaded cells on the undersides were fixed with 4% paraformaldehyde and stained with 0.1% crystal violet staining solution. The number of cells in 3 randomly selected fields was counted under a microscope. Each sample was analyzed three times in triplicate.

### 2.9. Wound Healing Assay

The wound healing assay was performed to investigate the alteration of cell motility and migration as previously described [29]. Briefly, cells were seeded in 6-well plates and cultured to a confluent monolayer. Then, an artificial wound was carefully created at 0 h using a standard 200 *μ*l pipette tip to scratch on the cell monolayer. Then, cells were washed with phosphate-buffered saline (PBS) to remove nonadherent cells, and wound closure was photographed (Olympus, Tokyo, Japan) at 18 h under serum-reduced medium. The percentage of wound closure was calculated using the ImageJ software (National Institutes of Health, Bethesda, Maryland). Each experiment was performed in triplicate and repeated three times.

### 2.10. EdU Incorporation Assay

The EdU incorporation assay was carried out according to the manufacturer's instruction (RiboBio, Guangzhou, China) as previously described [28]. Edu-positive cells were visualized and counted in 5 randomly selected fields under a fluorescent microscope (Zeiss, Jena, Germany). Each experiment was repeated three times.

### 2.11. Flow Cytometry Analysis

The proportion of the cells (TPC1/vector, TPC1/LOC100129940-N, KTC1/vector, and KTC1/LOC100129940-N) in the distinct cell cycle phase was determined by flow cytometry. At 60% confluence, cells were serum-deprived for 24 h for cell cycle synchronization and then incubated for 16 h in 10% FBS DMEM after synchronization. Thereafter, 1 × 10^6^ indicated cells were harvested and washed in ice-cold PBS, followed by fixation in 80% ethanol in PBS overnight at -20°C. Then, the cells were spun down and resuspended in cold PBS. The resuspended cells were incubated with 2 *μ*g/ml bovine pancreatic RNase (Sigma-Aldrich, St. Louis, MO), at 37°C for 30 min, followed by incubating cells for 20 min at room temperature with 20 *μ*g/ml of propidium iodide (PI, Sigma-Aldrich, St. Louis, MO). Flow cytometry data were acquired with a flow cytometer (Beckman Coulter, Hialeah, FL) and analyzed using FlowJo (TreeStar, Ashland, OR) [30].

### 2.12. Western Blotting (WB) Analysis

WB analysis was performed according to standard protocols as previously described [31]. RIPA lysis buffer (Thermo Fisher Scientific, Waltham, MA) was used to harvest the indicated cells. The bicinchoninic acid assay (BCA) (Thermo Fisher Scientific, Rockford, USA) was performed to assess the concentration of protein. Total cell lysates (30 *μ*g) were analyzed by sodium dodecyl sulfate-polyacrylamide gel electrophoresis (SDS-PAGE). After electroblotting to the PVDF membranes, the membranes were blocked with 5% nonfat milk for 1 h at room temperature. The membranes were then incubated with the primary antibodies anti-*β-*catenin antibody, anti-p84 antibody, anti-TCF4 antibody (Cell Signaling Technology, Beverly, MA), and anti-*α*-tubulin antibody (Sigma-Aldrich, St. Louis, MO). An enhanced chemiluminescence (ECL) detection kit (Pierce, Rockford, IL, USA) was used to detect the bands according to the manufacturer's instructions.

### 2.13. Statistical Analysis

The statistic differences between two groups were analyzed by paired, two-tailed Student's *t*-test. The one-way ANOVA test was used to analyze the differences between multiple comparisons. Descriptive values are expressed as mean ± standard deviation (SD). *P* < 0.05 was considered statistically significant. The SPSS 17.0 software (SPSS Inc., Chicago, IL) was used for statistical calculations.

## 3. Results

### 3.1. Identification of a Novel LOC100129940 Transcriptional Variant in PTC

**Figure 1:**
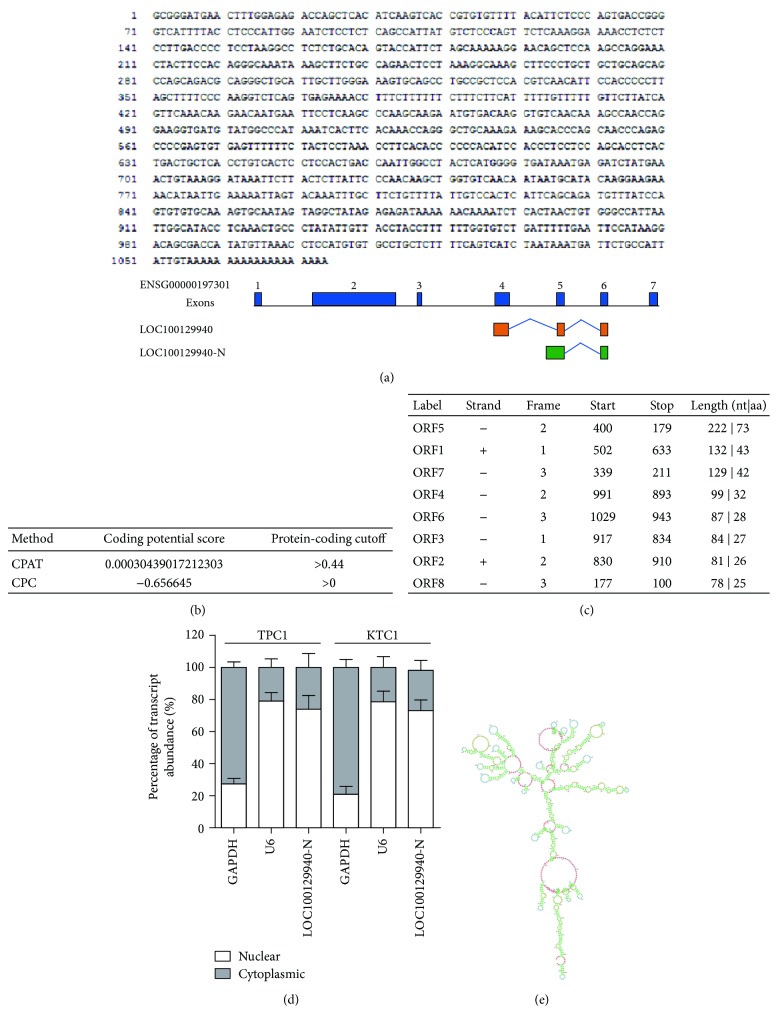
Identification of LOC100129940-N in PTC. (a) The sequence of the full length of LOC100129940-N (upper) and schematic representation of LOC100129940 and new transcript (designated as LOC100129940-N) (lower). (b) Analysis with the Coding-Potential Assessment Tool (CPAT) and the Coding Potential Calculator (CPC) indicated that the LOC100129940-N transcript has no protein-coding potential. (c) Analysis of the LOC100129940-N sequences by the ORF Finder. (d) The levels of GAPDH (cytoplasmic control), U6 (nuclear control), and LOC100129940-N were analyzed by qRT-PCR in nuclear and cytoplasmic fractions. Results derived from three independent experiments are expressed as mean ± SD. ^∗^ *P* < 0.05. (e) LOC100129940-N RNA folding is predicted using the program Mfold.

A previous study by Lan et al. showed that 3499 lncRNAs were differentially expressed between PTC tissues and adjacent nontumorous tissues using microarray [16]. Among these lncRNAs, 1192 were elevated and 2307 were downregulated. Of specific note, based on microarray data, Lan et al. performed qRT-PCR using RNA extracted from 57 pairs of PTC tissues and adjacent noncancerous thyroid tissues and confirmed that 5 lncRNAs, namely, TCONS 12 00010365, n386477, n340790, lnc-LLPH-2:1 (LOC100129940), and NR 003225.2, were significantly upregulated in malignant tissues [16]. Among them, the most upregulated lncRNA, n340790, has been demonstrated to play roles in the progression of thyroid cancer. We selected LOC100129940, the second upregulated lncRNA, for further study [32]. Initially, we performed 5′ and 3′ rapid amplification of cDNA ends (RACE) to explore the complete molecular structure of LOC100129940 (Figure 1(a) and Supplementary Figure 1). Intriguingly, based on the sequence information from 5′ and 3′ RACE, we identified a novel isoform of LOC100129940 (we named it as LOC100129940-N), but not the annotated one (LOC100129940) (Figure 1(a)). LOC100129940-N is a 1074-nt transcript with two exons and one intron. LOC100129940-N has a different 5′ end from the annotated one (Figure 1(a)). To determine if the obtained full-length transcript is truly a single transcript, RT-PCR was performed using specific primers targeting its 5′ end and 3′ end. As presented in Supplementary Figure 1, PCR products were detected, indicating that the cloned full-length LOC100129940-N is truly a single transcript. Next, analysis with the Coding-Potential Assessment Tool (CPAT) [33] and the Coding Potential Calculator (CPC) [34] indicated that the LOC100129940-N transcript has no protein-coding potential (Figure 1(b)). Sequence analysis of LOC100129940-N by the National Center for Biotechnology Information (NCBI) ORF Finder showed that it lacks long (*>*100 amino acids) ORFs (Figure 1(c)). Moreover, no canonical Kozak sequence was observed in LOC100129940-N, further suggesting the unlikelihood of translation of LOC100129940-N. We examined the subcellular localization of LOC100129940-N and found that it predominately resided in the nuclear fraction (Figure 1(d)). The RNA folding potential of the full-length LOC100129940-N was analyzed, and the predicted folding structure was presented in Figure 1(e). Together, we identify a novel lncRNA in PTC.

### 3.2. LOC100129940-N Is Upregulated in PTC

**Figure 2:**
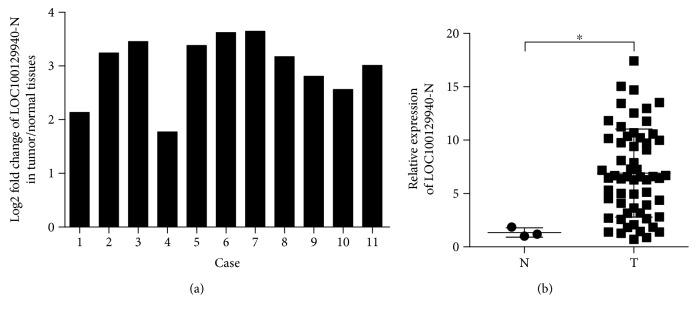
Upregulation of LOC100129940-N in PTC. (a) Analyses of the expression levels of LOC100129940-N in paired PTC and adjacent nontumorous tissues (*n* = 11). (b) Analyses of the expression levels of LOC100129940-N in 59 cases of PTC and 3 cases of normal thyroid tissues. ^∗^ *P* < 0.05.

We next investigated the expression of LOC100129940-N in PTC. Using the specific primer for LOC100129940-N, we analyzed its expression in 11 pairs of PTC specimens and matched nontumorous thyroid tissues. As presented in Figure 2(a), in comparison with the expression level in matched nontumorous thyroid tissues, the expression of LOC100129940-N was significantly elevated in PTC tissues. Next, we investigated the expression of LOC100129940-N in 59 cases of PTC specimens and 3 normal thyroid tissue samples. As shown in Figure 2(b), the increased abundance of LOC100129940-N was observed in 59 cases PTC as compared with 3 cases normal thyroid tissues. Together, these results demonstrate that LOC100129940-N is upregulated in PTC.

### 3.3. Overexpression of LOC100129940-N Enhances the Invasion, Migration, and Proliferation of PTC Cells

**Figure 3:**
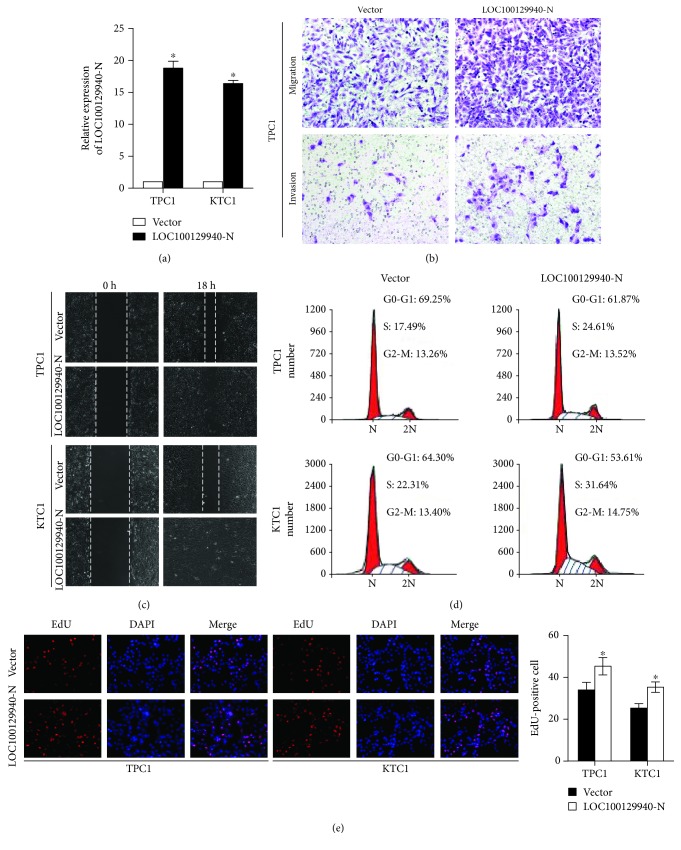
LOC100129940-N overexpression promotes the PTC cell invasion, migration, and proliferation. (a) Stable TPC1 and KTC1 cell lines overexpressing LOC100129940-N were established. ^∗^ *P* < 0.05. (b) Representative images of Transwell migration and invasion assays of the indicated cells. (c) Wound healing analyses of the indicated cells. Streaks were created with a tip, and the representative phase-contrast images of the extent of cell migration into the wounded area at the indicated time points are shown. (d) Flow cytometric determination of the proportion of the studied cells in distinct cell cycle phases. (e) Representative images and quantification of EdU incorporation assays. ^∗^ *P* < 0.05.

To investigate the potential biological functions of LOC100129940-N in PTC, we then established stable cell lines overexpressing LOC100129940-N, namely, TPC1/LOC100129940-N and KTC1/LOC100129940-N (Figure 3(a)). Initially, we investigated the role of LOC100129940-N in PTC cell invasion and migration by performing Matrigel-coated or uncoated Transwell assays. As shown in Figure 3(b) and Supplementary Figures 2a and 2b, ectopic overexpression of LOC100129940-N significantly promoted the capability of invasion and migration of PTC cells. Moreover, the significant increased closure of the wound was observed in cells with LOC100129940N overexpression as compared with vector-control cells (Figure 3(c) and Supplementary Figure 2c). Next, we also assessed the role of LOC100129940-N in PTC cell proliferation. Initially, the proportion of the indicated cells in the distinct cell cycle phase was determined by flow cytometry. As shown in Figure 3(d), flow cytometry analyses of the cell cycle distribution demonstrated that LOC100129940-N overexpression decreased the proportion of cells in the G1 phase as compared with that of vector-control cells. Moreover, the number of cells in the S phase was increased by overexpression of LOC100129940-N (Figure 3(d)). Meanwhile, the proproliferative role of LOC100129940-N was also demonstrated using EdU incorporation assays. As shown in Figure 3(e), LOC100129940-N significantly increased the number of EdU-positive cells in PTC cells. Taken together, these data indicate that LOC100129940-N promotes invasion, migration, and proliferation in PTC cells.

### 3.4. Silencing of LOC100129940-N Inhibits PTC Cell Invasion, Migration, and Proliferation

**Figure 4:**
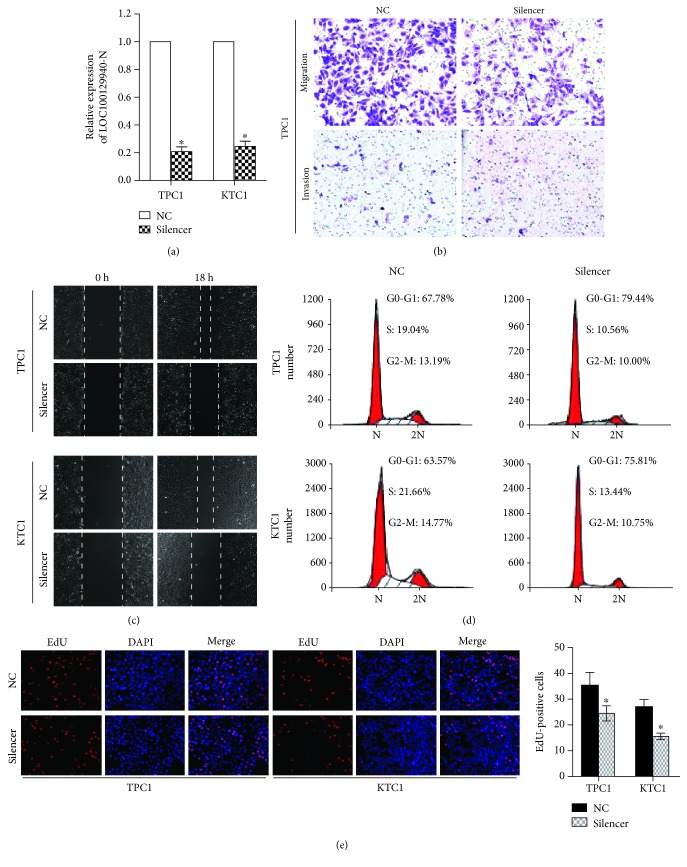
LOC100129940-N silencing suppresses the PTC cell invasion, migration, and proliferation. (a) Expression of LOC100129940-N was depleted in the indicated cells using the lncRNA Smart Silencer. ^∗^ *P* < 0.05. (b) Representative images of Transwell migration and invasion assays of the indicated cells. (c) Representative micrographs of the wound healing assay of the indicated cells. Wound closures were photographed at the indicated time after wounding. (d) Flow cytometric determination of the proportion of the studied cells in distinct cell cycle phases. (e) Representative images and quantification of EdU incorporation assays. ^∗^ *P* < 0.05.

To further investigate the biological role of LOC100129940-N in PTC cells, we depleted the expression of LOC100129940-N using the lncRNA Smart Silencer as described in Materials and Methods (Figure 4(a)). As shown in Figure 4(b) and Supplementary Figures 2d and 2e, Matrigel-coated or uncoated Transwell assays showed that LOC100129940-N silencing markedly reduced the invasive and migratory capability of PTC cells. As anticipated, depletion of LOC100129940-N in TPC1 and KTC1 cells inhibited the wound closure (Figure 4(c) and Supplementary Figure 2f). Next, the distribution of the cell cycle phase of the indicated cells was assessed by flow cytometry. As shown in Figure 4(d), the percentage of cells arrested in the G1 phase was increased in comparison to that of vector-control cells. Furthermore, LOC100129940-N silencing in PTC cells showed a substantial decrease in the S phase compared with that in vector-control cells (Figure 4(d)). In addition, the number of EdU-positive cells was significantly decreased in PTC cells with LOC100129940-N depletion (Figure 4(e)). These data, together with the results from gain-of-function experiments, strongly indicate that LOC100129940-N plays important roles in invasion, migration, and proliferation of PTC cells.

### 3.5. Wnt/*β*-Catenin Signaling Mediates LOC100129940-N-Induced Effects on PTC Cells

**Figure 5:**
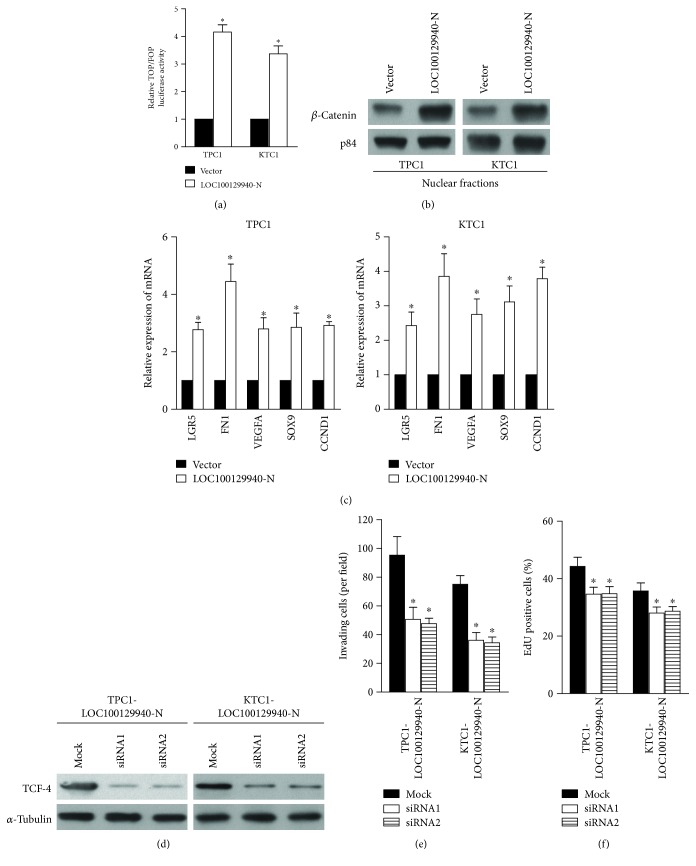
Overexpression of LOC100129940-N activates the Wnt/*β*-catenin signaling. (a) Luciferase assay for TOP/FOP reporters in the indicated cell. ^∗^ *P* < 0.05. (b) LOC100129940-N promoted nuclear translocation of *β*-catenin in the indicated cells. (c) The expression levels of LGR5, FN1, VEGFA, SOX9, and CCND1 were increased by the ectopic expression of LOC100129940-N. ^∗^ *P* < 0.05. (d) The expression of TCF4 in the indicated cells was analyzed by WB analysis. (e, f) Quantification of invading cells and EdU-positive cells in LOC100129940-N-overexpressing cells with silencing of TCF4. ^∗^ *P* < 0.05.

In exploring the mechanism underlying the roles of LOC100129940-N in cell proliferation and invasion, we initially investigated the roles of LOC100129940-N overexpression in MAPK-ERK and PI3/AKT signaling pathways and found no significant alterations in p-ERK and p-AKT levels in response to overexpression of LOC100129940-N in PTC cells (Supplementary Figure 3a). Next, we investigated the effects of overexpression of LOC100129940-N on NF-*κ*B signaling and Wnt/*β*catenin signaling using specific reporters. We found that the activity of NF-*κ*B signaling was not significantly altered in response to LOC100129940-N overexpression (Supplementary Figure 3b). Of note, the Top/Fop reporter assay demonstrated that TCF/LEF transcriptional activity was significantly elevated in TPC1 and KTC1 cells with LOC100129940-N overexpression as compared with vector-control cells. Moreover, the effect of LOC100129940-N on nuclear translocation of *β*-catenin, a character of Wnt/*β*-catenin activation, was detected. As expected, increased nuclear translocation of *β*-catenin was found in PTC cells with LOC100129940-N overexpression (Figure 5(b)). Additionally, the expression levels of several Wnt/*β*-catenin signaling downstream invasion- and proliferation-related molecules were confirmed. As shown in Figure 5(c), the expression of LGR5, FN1, VEGFA, SOX9, and CCND1 was upregulated by LOC100129940-N overexpression in PTC cells. We next investigated whether WNT/*β*-catenin signaling mediated the biological functions of LOC100129940-N on PTC cells. The inhibition of WNT/*β*-catenin signaling was achieved by silencing of TCF4 in TPC1 and KTC1 cells with LOC100129940-N overexpression (Figure 5(d)). LOC100129940-N-induced cell invasion and proliferation were abrogated by silencing of TCF4 (Figure 5(e)). Taken together, these data demonstrate that WNT/*β*-catenin signaling plays a vital role in the effects induced by LOC100129940-N in PTC cells.

### 3.6. Silencing of LOC100129940-N Inhibits WNT/*β*-Catenin Signaling

**Figure 6:**
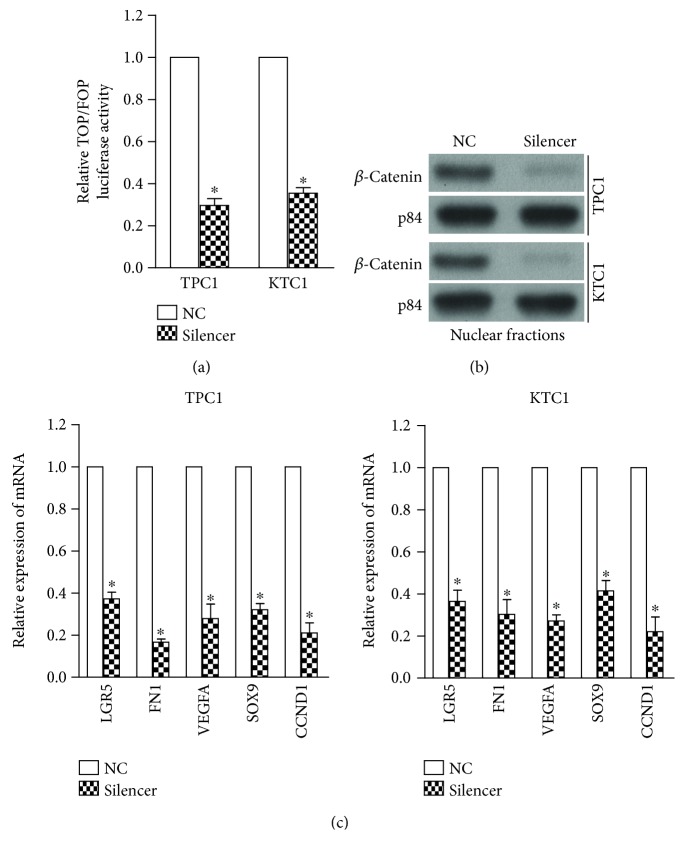
Depletion of LOC100129940-N inhibits the Wnt/*β*-catenin signaling. (a) Luciferase assay for TOP/FOP reporters in the indicated cell. ^∗^ *P* < 0.05. (b) Silencing of LOC100129940-N inhibited nuclear translocation of *β*-catenin in the indicated cells. (c) LOC100129940-N knockdown decreased the expression levels of LGR5, FN1, VEGFA, SOX9, and CCND1. ^∗^ *P* < 0.05.

To further investigate the role of LOC100129940-N in Wnt/*β*-catenin signaling, we assessed the effects of LOC100129940-N silencing on Wnt/*β*-catenin signaling in PTC cells. As shown in Figure 6(a), silencing of LOC100129940-N significantly suppressed the TCF/LEF transcriptional activity in TPC1 and KTC1 cells. Moreover, nuclear translocation of *β*-catenin was decreased in cells with LOC100129940-N silencing as compared with vector-control cells (Figure 6(b)). In addition, inhibition of LOC100129940-N decreased the expression of LGR5, FN1, VEGFA, SOX9, and CCND1 (Figure 6(c)). These further confirm the regulatory role of LOC100129940-N in Wnt/*β*-catenin signaling in PTC cells.

## 4. Discussion

In recent years, attention has turned increasingly toward the crucial roles of lncRNAs in cancer development and progression. Altered expression of lncRNAs in PTC has been established; however, the biological function and underlying mechanisms of many PTC-associated lncRNAs remain largely unknown. In the current study, we identified a new isoform of LOC100129940 and designated it as LOC100129940-N. We found that the expression of LOC100129940-N was upregulated in PTC as compared with the normal thyroid tissues. We further demonstrated that LOC100129940-N promotes PTC cell proliferation, invasion, and migration via activating Wnt/*β*-catenin signaling.

lncRNAs have been shown to play important roles in regulating malignant phenotypes of tumor cells. For example, lncRNA-TTN-AS1 plays an important role in esophageal squamous cell carcinoma (ESCC) cell proliferation and metastasis [35]. Moreover, Chen et al. demonstrated that lncRNA SNHG20 promotes non-small-cell lung cancer (NSCLC) cell proliferation and migration [36]. Several studies also demonstrated the important role of lncRNAs in PTC. Li et al. demonstrated that lncRNA HIT000218960 enhanced oncogenesis and tumor progression of PTC by upregulating the expression of the high-mobility group AT-hook 2 (HMGA2) gene [37]. Interestingly, plasma lncRNA GAS8-AS1 was demonstrated as a potential biomarker of PTC [38]. Our results indicate that LOC100129940-N may act as an oncogenic lncRNA in PTC. We examined the expression of LOC100129940-N in PTC and found that LOC100129940-N was upregulated in PTC. These indicate that LOC100129940-N may play an important role in PTC development and progression. Indeed, functional experiments showed that overexpression of LOC100129940-N enhanced the proliferation, invasion, and migration of PTC cells, whereas silencing of LOC100129940-N in PTC cells inhibited cell proliferation, invasion, and migration. Though the regulators involved in LOC100129940-N overexpression in PTC are still unknown, our data clearly demonstrated that it was upregulated in PTC in comparison with normal thyroid tissues.

To address the molecular mechanisms involved in LOC100129940-N-induced cell proliferation, invasion, and migration, we found that Wnt/*β*-catenin signaling may be involved in the biological effects of LOC100129940-N on PTC. Indeed, our data demonstrated that overexpression of LOC100129940-N in PTC cells led to the activation of Wnt/*β*-catenin signaling. In contrast, depletion of LOC100129940-N inhibited the Wnt/*β*-catenin signaling activity in PTC cells. Our findings concluded that the molecular basis for the oncogenic roles of LOC100129940-N in PTC might relate to the activation of Wnt/*β*-catenin signaling. Several lines of evidence are provided to support this conclusion. First, *β*-catenin-Tcf/Lef activity is upregulated and nuclear translocation of *β*-catenin is increased in response to ectopic overexpression of LOC100129940-N. Second, inhibition of Wnt/*β*-catenin signaling with Tcf4-specific siRNAs could abrogate the effects induced by LOC100129940-N. Third, the expression of LGR5, FN1, VEGFA, SOX9, and CCND1, which are important for cell invasion and growth, was elevated by LOC100129940-N overexpression. Fourth, silencing of LOC100129940-N suppressed the activation of Wnt/*β*-catenin signaling and the expression of downstream target genes.

Hyperactivation of Wnt/*β*-catenin signaling has been demonstrated in various malignant diseases, including thyroid cancer [39, 40]. Nevertheless, the mechanisms involved in the activation of Wnt/*β*-catenin signaling in PTC are not well understood. Here, we found that LOC100129940-N activated the Wnt/*β*-catenin signaling and thereby enhanced the malignant phenotypes of PTC cells. However, the mechanisms underlying the relationship between LOC100129940-N and Wnt/*β*-catenin signaling need to be further explored.

## 5. Conclusion

Collectively, our data demonstrate that LOC100129940-N is upregulated in PTC and promotes activation of Wnt/*β*-catenin signaling. Through the activation of Wnt/*β*-catenin signaling and upregulation of downstream target genes, LOC100129940-N promotes PTC cell proliferation, invasion, and migration. These results implicate that targeting LOC100129940-N could be a potential therapeutic strategy.

## Data Availability

The data used to support the findings of this study are included within the article.

## Supplementary Materials

Supplementary Materials Supplementary Table 1: primers used in the quantitative RT-PCR assays. Fig. 1: the images of PCR products from the 5′-RACE and 3′-RACE procedure. Fig. 2: LOC100129940-N regulates the PTC cell invasion and migration. a Representative images of Transwell migration and invasion assays of the indicated cells. b Quantification of the indicated invading or migrating cells analyzed by Matrigel-coated or noncoated Transwell assays, respectively. c Quantification of wound closures of the indicated cells. d Representative images of Transwell migration and invasion assays of the indicated cells. e Quantification of the indicated invading or migrating cells. f Quantification of wound closures of the indicated cells. For b, c, e, and f, results derived from three independent experiments are expressed as mean ± SD. ^∗^ *P* < 0.05. Fig. 3: the effects of LOC100129940-N on ERK, AKT, and NF-*κ*B signaling. a No significant alterations in p-ERK and p-AKT levels in response to overexpression of LOC100129940-N in PTC cells. b Luciferase assay for NF-*κ*B reporters in the indicated cells.
