# Supplementary material for: Long Noncoding RNA LOC100129940-N Is Upregulated in Papillary Thyroid Cancer and Promotes the Invasion and Progression
Source: Int J Endocrinol. 2019 Apr 7;2019:7043509. doi: 10.1155/2019/7043509 (PMC6476119; doi:10.1155/2019/7043509)

Supplementary Table 1: Primers used in the quantitative RT-PCR assays.

| **Primers** | **Sequences (5’ to 3’)** |
| --- | --- |
| LGR5-Forward | GTTTCCCGCAAGACGTAACT |
| LGR5-Reverse | CAGCGTCTTCACCTCCTACC |
| FN1-Forward | ACCTCGGTGTTGTAAGGTGG |
| FN1-Reverse | CCATAAAGGGCAACCAAGAG |
| SOX9-Forward | GTAATCCGGGTGGTCCTTCT |
| SOX9-Reverse | GACGCTGGGCAAGCTCT |
| FGF18-Forward | CTTACGGCTCACATCGTCC |
| FGF18-Reverse | ACTTCCTGCTGCTGTGCTTC |
| VEGFA-Forward | AGCTGCGCTGATAGACATCC |
| VEGFA-Reverse | CTACCTCCACCATGCCAAGT |
| LOC100129940-N-Forward | CAACATTCCACCCCCTTAGC |
| LOC100129940-N-Reverse | CATTCTTGCTTGGGCTTGAG |


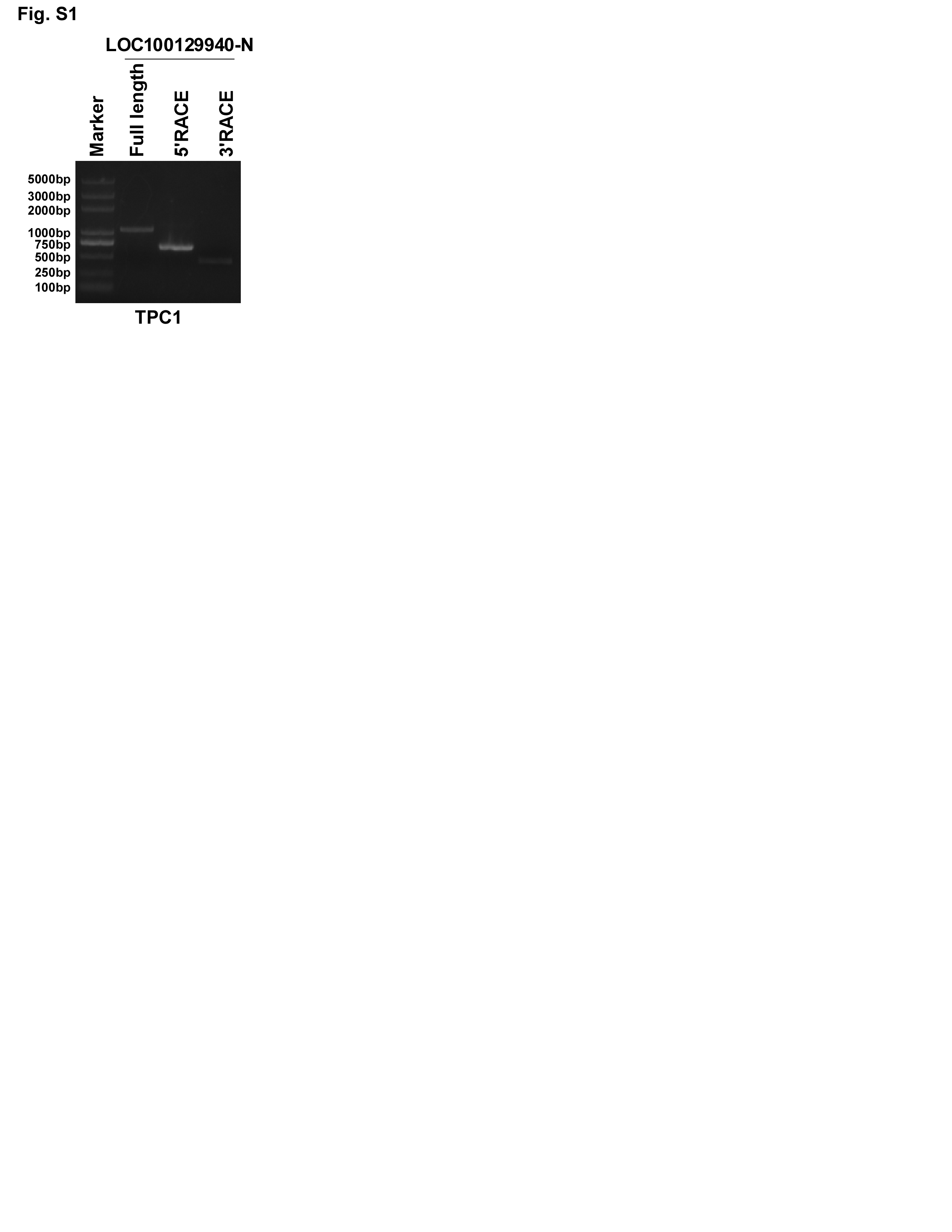


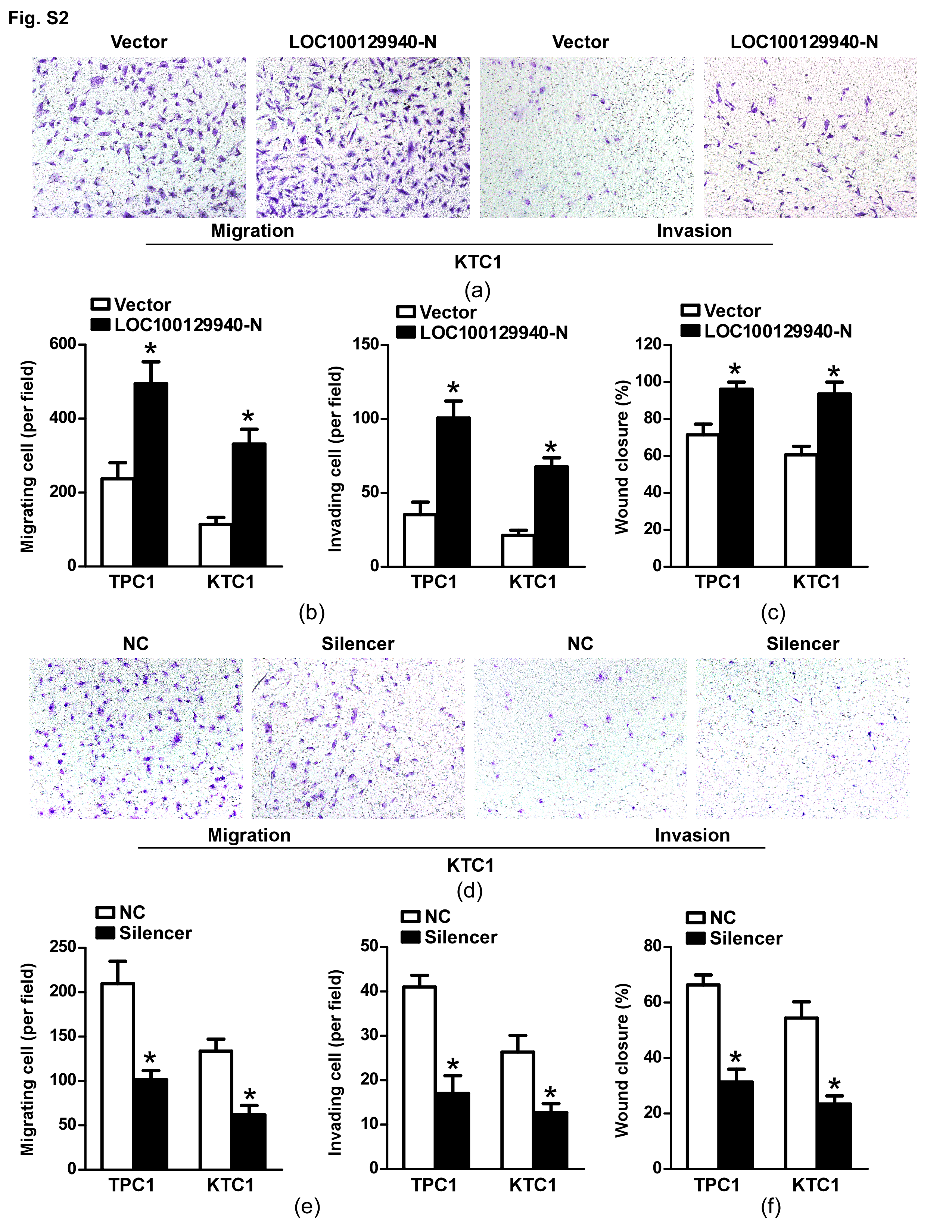


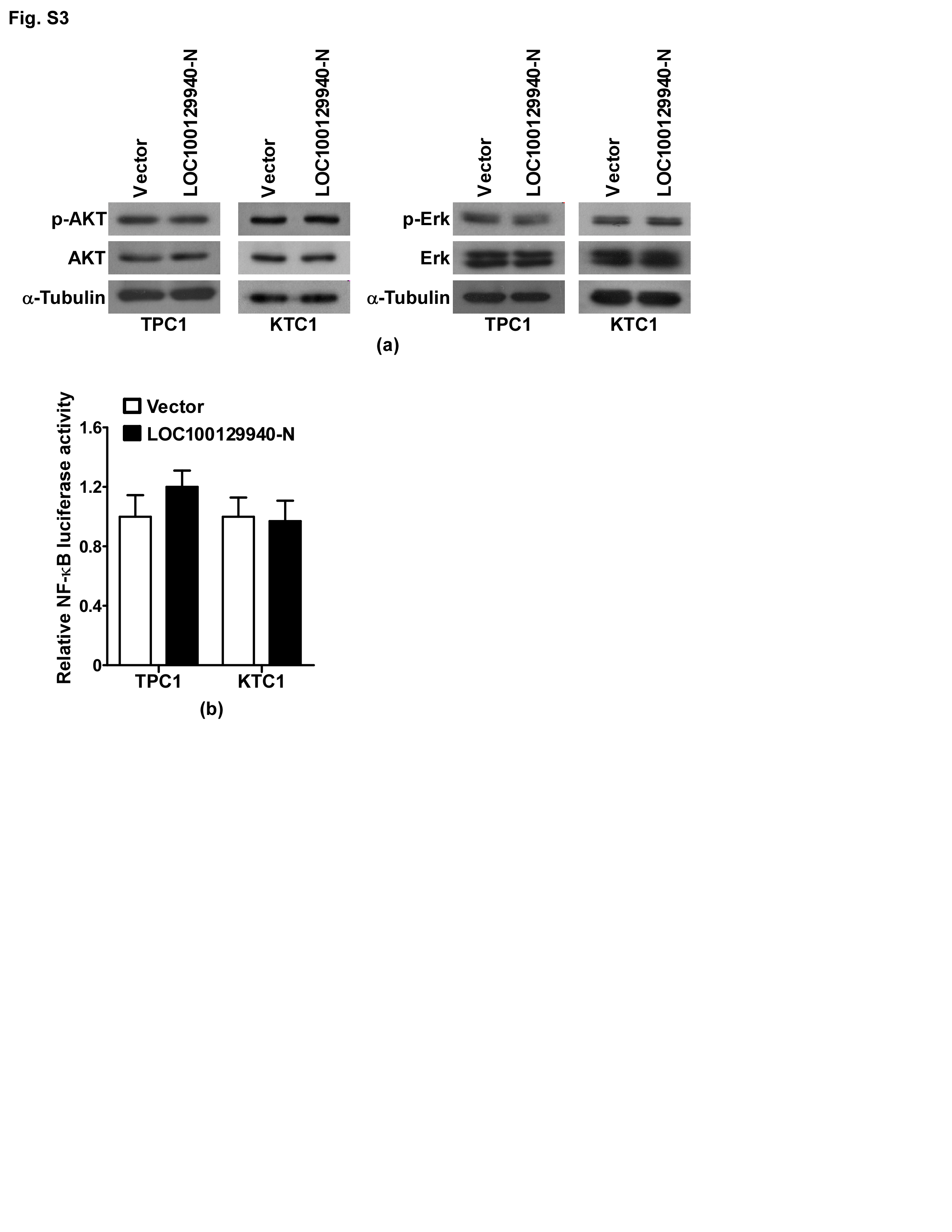

Supplement: Supplementary Materials — Supplementary Table 1: primers used in the quantitative RT-PCR assays. Fig. 1: the images of PCR products from the 5′-RACE and 3′-RACE procedure. Fig. 2: LOC100129940-N regulates the PTC cell invasion and migration. a Representative images of Transwell migration and invasion assays of the indicated cells. b Quantification of the indicated invading or migrating cells analyzed by Matrigel-coated or noncoated Transwell assays, respectively. c Quantification of wound closures of the indicated cells. d Representative images of Transwell migration and invasion assays of the indicated cells. e Quantification of the indicated invading or migrating cells. f Quantification of wound closures of the indicated cells. For b, c, e, and f, results derived from three independent experiments are expressed as mean ± SD. ∗ P < 0.05. Fig. 3: the effects of LOC100129940-N on ERK, AKT, and NF-κB signaling. a No significant alterations in p-ERK and p-AKT levels in response to overexpression of LOC100129940-N in PTC cells. b Luciferase assay for NF-κB reporters in the indicated cells. [file 7043509.f1.docx]
